# Cardiac medication prescribing and adherence after acute myocardial infarction in Chinese and South Asian Canadian patients

**DOI:** 10.1186/1471-2261-11-56

**Published:** 2011-09-18

**Authors:** Emily J Lai, Maja Grubisic, Anita Palepu, Hude Quan, Kathryn M King, Nadia A Khan

**Affiliations:** 1 Department of Medicine, University of British Columbia, 10th floor - 2775rel S Laut., Vancouver, BC V5Z 1M9, Canada; 2 Center for Health Evaluation and Outcomes Sciences, St. Paul's Hospital, 620B - 1081 Burrard St., Vancouver, BC V6Z 1Y6, Canada; 3 Department of Community Health Sciences, University of Calgary, TRW Bldg 3rd floor - 3280 Hospital Dr NW, Calgary, AB T2N 4Z6, Canada

**Keywords:** medication adherence, acute myocardial infarction, ethnicity

## Abstract

**Background:**

Failure to adhere to cardiac medications after acute myocardial infarction (AMI) is associated with increased mortality. Language barriers and preference for traditional medications may predispose certain ethnic groups at high risk for non-adherence. We compared prescribing and adherence to ACE-inhibitors (ACEI), beta-blockers (BB), and statins following AMI among elderly Chinese, South Asian, and Non-Asian patients.

**Methods:**

Retrospective-cohort study of elderly AMI survivors (1995-2002) using administrative data from British Columbia. AMI cases and ethnicity were identified using validated ICD-9/10 coding and surname algorithms, respectively. Medication adherence was assessed using the 'proportion of days covered' (PDC) metric with a PDC ≥ 0.80 indicating optimal adherence. The independent effect of ethnicity on adherence was assessed using multivariable modeling, adjusting for socio-demographic and clinical characteristics.

**Results:**

There were 9926 elderly AMI survivors (258 Chinese, 511 South Asian patients). More Chinese patients were prescribed BBs (79.7% vs. 73.1%, p = 0.04) and more South Asian patients were prescribed statins (73.5% vs. 65.2%, p = 0.001). Both Chinese (Odds Ratio [OR] 0.53; 95%CI, 0.39-0.73; p < 0.0001) and South Asian (OR 0.78; 95%CI, 0.61-0.99; p = 0.04) patients were less adherent to ACEI compared to Non-Asian patients. South Asian patients were more adherent to BBs (OR 1.3; 95%CI, 1.04-1.62; p = 0.02). There was no difference in prescribing of ACEI, nor adherence to statins among the ethnicities.

**Conclusion:**

Despite a higher likelihood of being prescribed evidence-based therapies following AMI, Chinese and South Asian patients were less likely to adhere to ACEI compared to their Non-Asian counterparts.

## Background

Acute myocardial infarction (AMI) is one of the leading causes of death across multiple ethnic groups in North America. Landmark clinical trials established the efficacy of medications in reducing morbidity and mortality associated with AMI [1-3]. The morbidity and mortality benefits observed in these trials were largely among patients who were highly adherent. However, in real-world settings, typical adherence rates for prescribed medications are 50%, and are even lower in developing countries [4,5]. Medication non-adherence is associated with substantial worsening of disease, increased health care costs, and death [6-9]. From re-hospitalizations to lost workdays, the collective economic burden of non-adherence is estimated to be over $100 billion per year.

Non-adherence is a multidimensional phenomenon, affected by socio-economic status, health systems, disease states, pharmacological therapies, and patient beliefs [5]. Whether patient ethnicity plays a role in medication adherence is unclear [5,10]. To date, the literature yields variable results [11-14] with little data on medication adherence in Chinese and South Asian populations, the largest, and fastest growing, ethnic groups in North America. Language barriers, mistrust of Western medicine, and preference for traditional therapies could adversely impact medication adherence in these groups. Furthermore, different ethnicities may react differently to the medications. For example, Asian patients have been noted to have a greater risk for adverse effects from ACEI [15]. Therefore, we compared prescribing and adherence to evidence-based therapies [ACE inhibitors (ACEI), beta-blockers (BB), and HMG-CoA reductase inhibitors (statin)] using a large multi-ethnic cohort of elderly Chinese, South Asian, and Non-Asian survivors of AMI.

## Methods

Our research conformed to the Helsinki Declaration and to local legislation. Ethics approval was obtained from The University of British Columbia Providence Health Care Research Ethics Board.

### Data Sources

**Figure 1:**
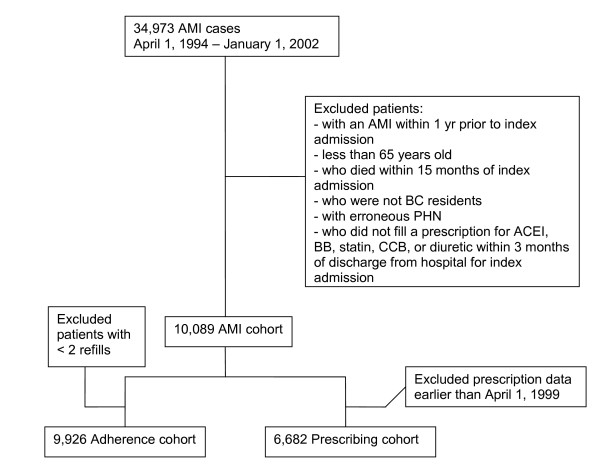
**Patient Flow Diagram**. ACEI = angiotensin converting enzyme inhibitor, AMI = acute myocardial infarction, BB = beta-blocker, BC = British Columbia, CCB = calcium channel blocker, PHN = personal health number, statin = HMG-CoA reductase inhibitor.

We used hospital discharge administrative data in British Columbia (BC), Canada to identify index cases of AMI between April 1^st^, 1994 and January 1^st^, 2002 (Figure 1). This database contains information on co-morbid conditions, hospital characteristics, and demographic data. Since Canada has a universal health insurance system, this data is available for all hospitalized patients in the province.

Medication prescription (for any of ACEI, BB, or statin) was determined by linkage to the BC Pharmacare prescription database. These medication classes were selected because of their proven mortality benefit in secondary prevention of cardiovascular events [1-3,16-19]. The Pharmacare database contains records of all outpatient prescriptions filled in BC by residents aged 65 years or older including date of prescription fill and days of medication supplied. Previous studies demonstrate excellent accuracy with prescription claims databases [0.7% error rate] [20]. By restricting our analysis to patients aged 66 years and older, we minimize the effects of patient costs on adherence as these individuals pay a deductible on medications up to Cdn$200/year, which was increased to Cdn$275/year on January 1^st^, 2002. All medication costs above this deductible are paid by Pharmacare.

### Study population

The cohort consisted of patients aged 66 years or older who were discharged from hospital with a most responsible diagnosis of AMI. To identify index AMI cases, we used the International Classification of Diseases (ICD9/ICD10) coding algorithms for hospital administrative data [ICD9 410.x; ICD10: I21.x]. These coding algorithms for AMI have been extensively validated against multi-centre chart audits [21-23]. We only included patients who survived at least 1 year and 3 months after the hospital admission, to allow for a sufficient time period to calculate medication adherence and excluded non-BC residents.

### Identification of ethnicity

Ethnicity is the common and/or inherited traits shared by people of the same race, ancestry, background and/or culture [24,25]. As self-reported ethnicity is not available in administrative databases, we used surname algorithms to categorize patient ethnicity as Chinese (from China, Taiwan or Hong Kong) or South Asian (from Pakistan, India or Bangladesh). The remaining patients will be referred to as 'Non-Asian' although 7% of this non-Chinese, non-South Asian group is a visible minority according to the Canadian Census [26]. To identify patients of Chinese descent, we used Quan's surname algorithm that has a sensitivity of 78%, a specificity of 99.7% and a positive predictive value of 81% compared to self-reported ethnicity using the Canadian Community Health Survey [27]. The Nam Pehchan surname algorithm has a 90-94% sensitivity, a 99.4% specificity and a positive predictive value of 63-96% for determining South Asian ethnicity [28,29].

### Prescribing

Since the Pharmacare database only includes data on prescription medications, we limited our selections to ACEIs, BBs, statins, calcium-channel blockers (CCB), and diuretics. Calcium-channel blockers and diuretics were included to contrast the prescribing of cardiac medications with proven and non-proven mortality benefit. Angiotensin II receptor blockers (ARBs) were not included in our analysis as these agents were not part of post-AMI guidelines during the study period. We collected data on prescribing, within the 3 months prior to AMI and at 1 year after AMI. Since prescribing patterns change over time, we restricted our collection period to April 1^st^, 1999 to March 31^st^, 2003 to better reflect more recent prescribing practices.

### Assessment of adherence

We used the 'proportion of days covered' (PDC), a commonly used metric for evaluating medication adherence. The PDC represents the number of days a patient had a medication available, divided by the days observed [6,30]. Patients had to obtain a supply of medication within 3 months of hospital discharge. We used prescription data from April 1^st^, 1994 to March 31^st^, 2003 to ensure we had an adequate sample size. To better reflect long-term medication adherence, we used an observation period of 1 year after the first-filled prescription [6]. A previous study of AMI patients demonstrated longer assessments of adherence were not significantly different from a one-year measurement [6]. We calculated the PDC for a single class of medication, as well as the PDC for any one of ACEI, BB, or statin prescriptions, since our interest was in adherence to *any or all *proven therapies. We defined adherence as a PDC of ≥ 80%, and suboptimal adherence as a PDC < 80%. The 80% cut-point is similar to that used in other medication adherence studies and is associated with mortality benefit after AMI compared to other levels of adherence [6,30].

### Other variables

We adjusted for several factors that have previously been shown to affect medication adherence, including previous use of the drug and number of medications (see Table 1 for list) [6,30-33]. We also adjusted for the number of hospital re-admissions within the year post-AMI as this reflects periods where patients are less likely to fill prescriptions [5,6]. To control for severity of illness on admission between ethnic groups, we used the clinical and demographic variables from the validated Ontario AMI mortality prediction rule [34].

**Table 1:** Patient characteristics according to ethnicity

Characteristic	Chinese	South Asian	Non-Asian	p-value
	**N = 258**	**N = 511**	**N = 9157**	

Age, n (%)				

65-69 years	66 (25.6)	167 (32.7)	2297 (25.1)	0.0006

70-74 years	63 (24.4)	143 (28)	2330 (25.5)	0.40

75-79 years	62 (24)	104 (20.4)	2111 (23.1)	0.34

≥ 80 years	67 (26)	97 (19)	2419 (26.4)	0.001

Female, n (%)	93 (36.1)	204 (39.9)	3718 (40.6)	0.33

Income, n (%) *				

< $30,569	83 (32.2)	129 (25.2)	2165 (23.6)	< 0.0001

$30,570-43,147	78 (30.2)	138 (27)	1735 (19)	< 0.0001

$43,148 - 54,103	36 (14)	110 (21.5)	1642 (17.9)	< 0.0001

$54,104 - 68,206	23 (8.9)	62 (12.1)	1609 (17.6)	< 0.0001

$68,207 - 221,991	35 (13.6)	55 (10.8)	1559 (17)	< 0.0001

> 50km to hospital, n (%)	12 (4.7)	74 (14.5)	3181 (34.7)	< 0.0001

Number of hospitalizations, mean (SD)	0.18 (0.49)	0.31 (0.66)	0.3 (0.64)	< 0.0001

Total # of medications^†^, mean (SD)	7.51 (3.81)	8.18 (3.9)	7.32 (3.63)	0.011

Prior use of ACEI, BB, or statin^‡^, n (%)	109 (42.3)	188 (36.8)	3376 (36.9)	0.21

Comorbidities, n (%)				

Diabetes	67 (26)	152 (29.8)	1526 (16.7)	< 0.0001

CHF	58 (22.5)	110 (21.5)	1570 (17.2)	0.004

Cardiac	40 (15.5)	60 (11.7)	1392 (15.2)	0.10

PAD	7 (2.7)	4 (0.8)	342 (3.7)	0.0016

Hypertension	98 (38)	160 (31.3)	2402 (26.2)	< 0.0001

Cerebrovascular	12 (4.7)	9 (1.8)	203 (2.2)	0.03

Kidney disease	15 (5.8)	16 (3.1)	180 (2)	< 0.0001

Abbreviations: ACEI = angiotensin-converting-enzyme inhibitor; BB = beta-blocker; CHF = congestive heart failure; PAD = peripheral arterial disease; PDC = proportion of days covered

* Missing socio-economic status data was < 4.8% (1.6% Chinese, 3.4% South Asians, 4.9% Non-Asians)

^† ^Within 3 months of discharge from index admission date

^‡ ^Within 3 months prior to index admission date

### Statistical analysis

Baseline characteristics were compared between ethnic groups using chi-square testing for categorical variables and ANOVA for continuous variables. Missing values, found in measures of socio-economic status quintile (< 4.8%), were excluded from the analysis (see Table 1). Multivariable logistic regression models, adjusting for age, sex, residential distance from hospital, income quintile, admission year, number of baseline medications, number of re-admissions to hospital, prior use of same medication, and comorbid conditions from the Ontario AMI prediction rule, were constructed to examine the independent relationship between adherence (PDC ≥ 80%) and ethnicity. Logistic regression model assumptions were satisfied. Statistical significance was defined as a 2-tailed p < 0.05. All analyses were performed using SAS statistical software version 9.1 (SAS Institute Inc, Cary, NC).

## Results

### Baseline characteristics

Of 9926 patients who met inclusion criteria, 258 (2.6%) were Chinese, 511 (5.1%) were South Asian, and 9157 (92.3%) were categorized as Non-Asian. Table 1 illustrates baseline socio-demographic and clinical characteristics between the three ethnic groups. Chinese and South Asian patients tended to reside in urban areas and comprised a larger proportion of the lower income quintiles than Non-Asian patients. There were more Chinese and South Asian patients with diabetes, congestive heart failure, kidney disease, hypertension, and more Chinese patients with cerebrovascular disease. Both Chinese and South Asian patients were prescribed a greater number of total medications within 3 months of hospital discharge.

### Prescribing of evidence-based therapies

In all ethnic groups, there were significant increases in prescriptions for evidence-based therapies after discharge from hospital for AMI (Table 2). However, despite the high risk of re-infarction, 25% of patients did not fill a prescription for any evidence-based therapies. Compared to Non-Asian patients, Chinese patients were more likely prescribed BBs, while South Asian patients were more likely prescribed statins. There was no significant difference in ACEI prescribing between the ethnic groups. There was also no difference in prescribing of CCBs between the ethnic groups. While more Non-Asian patients were prescribed diuretics prior to AMI, there was no difference in diuretic prescriptions between the ethnic groups post-AMI.

**Table 2:** Medication prescribing according to ethnicity 3 months prior to AMI and 1 year post AMI, n (%)

Medication	3 months prior to AMI				1 year post AMI			
	
	Chinese (n = 150)	South Asian (n = 224)	Non-Asian (n = 3697)	p-value	Chinese (n = 197)	South Asian (n = 370)	Non-Asian (n = 6115)	p-value
ACEI	66 (44)	86 (38.4)	1586 (42.9)	0.39	157 (79.7)	293 (79.2)	4621 (75.6)	0.13

BB	56 (37.3)	66 (29.5)	1134 (30.7)	0.2	157 (79.7)	286 (77.3)	4472 (73.1)	0.03*

Statin	40 (26.7)	69 (30.8)	973 (26.3)	0.34	132 (67)	272 (73.5)	3987 (65.2)	0.004^†^

ACEI, BB, or Statin	110 (73.3)	154 (68.8)	2641 (71.4)	0.59	194 (98.5)	360 (97.3)	5867 (95.9)	0.09

CCB	61 (40.7)	84 (37.5)	1312 (35.5)	0.37	66 (33.5)	131 (35.4)	1856 (30.4)	0.09

Diuretic	42 (28)	76 (33.9)	1568 (42.4)	< 0.001	104 (52.8)	189 (51.2)	2990 (48.9)	0.42

Abbreviations: ACEI = angiotensin-converting-enzyme inhibitor; AMI = acute myocardial infarction; BB = beta-blocker; CCB = calcium-channel blocker

* For BBs: Chinese vs. Non-Asian, p = 0.04; South Asian vs. Non-Asian, p = 0.08

^† ^For statins: Chinese vs. Non-Asian, p = 0.6; South Asian vs. Non-Asian, p = 0.001

### Adherence to evidence-based therapies

The majority (79.9%) of patients were adherent to at least one of the three evidence-based therapies (Table 3). In the unadjusted analysis, there was no significant difference between the ethnic groups for adherence to any one of ACEI, BB, or statin medication (82.6% South Asian vs. 76.4% Chinese vs. 79.9% Non-Asian patients). Examining individual classes of medications, there was a smaller proportion of Chinese patients adhering to ACEI and a larger proportion of South Asian patients adhering to BBs relative to Non-Asian patients. Adherence to statins was similar across all ethnic groups. Adherence to CCBs and diuretics was also similar across all ethnicities, although adherence rates for diuretics were low in all groups.

**Table 3:** Adherence to cardiac medications (PDC ≥ 80%) according to ethnicity and medication

Medication	Chinese	South Asian	Non-Asian	p-value	Adj. OR (95%CI)*		Adj. OR (95%CI)*	
					
					Chinese vs. Non-Asian	p-value	South Asian vs. Non-Asian	p-value
ACEI, %	63.5	71.4	75	< 0.001	0.53 (0.39-0.73)	< 0.001	0.78 (0.61-0.99)	0.04

BB, %	54.1	63.4	56.4	0.02	0.85 (0.63-1.15)	0.29	1.3 (1.04-1.62)	0.02

Statin, %	80.8	85.4	81	0.19	0.83 (0.52-1.35)	0.46	1.26 (0.88-1.79)	0.21

CCB, %	64	68.4	65.5	0.73	0.84 (0.51-1.38)	0.49	1.07 (0.74-1.53)	0.73

Diuretic, %	35.9	33.8	39.5	0.18	0.84 (0.55-1.28)	0.41	0.73 (0.54-0.99)	0.04

ACEI, BB, or statin, %	76.4	82.6	79.9	0.12	0.70 (0.51-0.95)	0.02	1.01 (0.79-1.29)	0.92

Abbreviations: ACEI = angiotensin-converting-enzyme inhibitor; Adj. OR = adjusted odds ratio; BB = beta-blocker; CCB = calcium-channel blocker; PDC = proportion of days covered

* odds ratios adjusted for age, gender, residential distance from hospital, socio-economic status, admission year, number of baseline medications, number of re-admissions to hospital, prior use of same medication, and comorbidities

In the adjusted analysis, Chinese patients were less likely to adhere to ACEI, compared to Non-Asians, [OR 0.53; 95%CI: 0.39-0.73]. Chinese patients, overall, were also less likely to be adherent to any of ACEI, BB, or statin medication [OR 0.70; 95%CI: 0.51-0.95]. Compared to Non-Asians, South Asians were less likely to be adherent to ACEI [OR 0.78; 95%CI: 0.61-0.99] but more likely to be adherent to BBs [OR 1.3; 95%CI: 1.04-1.62]. Among the medications with less evidence for cardio-protection, there was no significant difference in adherence to CCBs, but South Asian patients were less likely to adhere to diuretics compared to Non-Asian patients.

## Discussion

In this study, elderly Chinese and South Asian patients were as or more likely to be prescribed evidence-based therapies following AMI compared to their Non-Asian counterparts. However, adherence varied by medication class in the ethnic groups.

Overall prescribing rates for secondary prevention of AMI were poor for statin medications (68%) but higher for ACEI and BB medications (77-78%). Overall prescribing of these medications was similar to those in other studies [6,35]. Appropriately, we saw a decrease in prescribing for CCBs. We found that Chinese and South Asian patients were more likely to be prescribed BBs and statins compared to Non-Asian patients. Reasons for this are unclear; prescribing physicians may consider Chinese and South Asian patients to be at higher cardiac risk, necessitating more aggressive management. Studies demonstrate that South Asian patients, for example, are more or just as likely to receive invasive cardiovascular procedures following AMI, compared to their Non-Asian counterparts [36]. Alternatively, Chinese and South Asian patients may have been more likely to fill their prescriptions once discharged from hospital compared to Non-Asian patients. Non-Asian patients tended to reside outside of urban areas where access to medical follow-up was perhaps more limited, potentially resulting in fewer opportunities to fill prescriptions.

This study found that elderly Chinese patients were less likely to adhere to any evidence based therapy following AMI relative to Non-Asian patients. To our knowledge, this is the first study evaluating the adherence to secondary prevention medications following AMI in Chinese and South Asian patients. In addition to the factors associated with non-adherence found in the general population, non-adherence in these ethnic populations may be further amplified by language barriers [37-40] and differences in health literacy [40-42] among ethnocultural groups. Furthermore, ethnocultural patients may have a preference for alternative or natural therapies [43-45], and some may perceive that antihypertensive therapy is not beneficial [46]. Furthermore, differences in health beliefs and strong Eastern views of care (e.g. viewing disease as a result of fate and avoidance of medical visits) are associated with poor adherence to treatment recommendations [47]. Intriguingly, even within the same class of medication, we found that adherence varied by patient ethnicity with the greatest proportion of suboptimal adherence for ACEI in both Chinese and South Asian patients. This observation raises suspicion that the differences in adherence may be, at least in part, attributed to greater adverse effect profiles within these ethnic groups. In a systematic review of cardiovascular drug utilization, ACEI-induced cough was more prevalent in Asian patients than in the general population, although South Asian patients were not studied separately [15]. Adherence to statins was similar across ethnic groups despite the fact that, in post-marketing surveillance, rosuvastatin was associated with greater statin-induced myopathy in Asian patients [48].

This study had several limitations. First, we only used a proxy measurement for adherence using prescriptions claims data. Although this approach does not ensure that the medications were ingested, prescription claims data highly correlates with home inventory pill counts, as well as serum measures of drug presence [49-51]. As with all observational studies, we were limited by residual confounders since we were unable to assess other factors associated with non-adherence such as dementia and depression. Similarly, we did not have access to other clinical information such as left ventricular function or creatinine, but we attempted to adjust for some of the clinical variables by including the diagnosis of CHF or kidney disease, for example, in Table 1. We did not include Angiotensin receptor blockers (ARBs) in our analysis. These agents have become more widely used since the study period, and are often prescribed for patients who experience negative side effects from ACEI, thereby potentially impacting adherence to any renin-angiotensin-aldosterone agent (either ACEI or ARBs). Finally, we investigated medication adherence among major ethnic groups in Canada who were elderly; these results may not be generalized to other ethnic groups or to younger patients within these groups.

## Conclusions

Compared to their Non-Asian counterparts, South Asian and Chinese elderly patients are just as likely or more likely to receive proven secondary prevention therapies. However, in this cohort of high cardiac risk patients, 25% of patients did not fill a prescription for these therapies, suggesting greater need to improve prescribing in all ethnic groups following AMI. Chinese patients were less likely to be adherent to any secondary prevention medication and specifically, ACEI therapy. South Asian patients were also less likely to be adherent to ACEI therapy relative to their Non-Asian counterparts. This study identifies an at-risk group of patients that require aggressive monitoring, follow-up and support to optimize adherence. Future studies evaluating underlying cultural barriers to adherence are needed to develop culturally-tailored interventions to improve adherence. The disproportionately lower adherence to ACEI in South Asian and Chinese patients, raises suspicion that both of these groups may suffer greater adverse effects associated with ACEI.

## Competing interests

The authors declare that they have no competing interests.

## Authors' contributions

All authors listed have contributed sufficiently to the project to be included as authors. All authors contributed to the acquisition and analysis/interpretation of data, as well as revised the manuscript. In addition, EJL drafted the manuscript and NAK contributed to the conception and design. All authors have given final approval of the version to be published.

## Pre-publication history

The pre-publication history for this paper can be accessed here:

http://www.biomedcentral.com/1471-2261/11/56/prepub
